# Divergent Evolution of a Protein–Protein Interaction Revealed through Ancestral Sequence Reconstruction and Resurrection

**DOI:** 10.1093/molbev/msaa198

**Published:** 2020-08-04

**Authors:** Louise Laursen, Jelena Čalyševa, Toby J Gibson, Per Jemth

**Affiliations:** 1 Department of Medical Biochemistry and Microbiology, Uppsala University, Uppsala, Sweden; 2 Structural and Computational Biology Unit, European Molecular Biology Laboratory, Heidelberg, Germany; 3 Faculty of Biosciences, Collaboration for Joint PhD Degree between EMBL and Heidelberg University

**Keywords:** protein–protein interaction, ancestral sequence reconstruction, protein evolution, CRIPT, PSD-95, DLG

## Abstract

The postsynaptic density extends across the postsynaptic dendritic spine with discs large (DLG) as the most abundant scaffolding protein. DLG dynamically alters the structure of the postsynaptic density, thus controlling the function and distribution of specific receptors at the synapse. DLG contains three PDZ domains and one important interaction governing postsynaptic architecture is that between the PDZ3 domain from DLG and a protein called cysteine-rich interactor of PDZ3 (CRIPT). However, little is known regarding functional evolution of the PDZ3:CRIPT interaction. Here, we subjected PDZ3 and CRIPT to ancestral sequence reconstruction, resurrection, and biophysical experiments. We show that the PDZ3:CRIPT interaction is an ancient interaction, which was likely present in the last common ancestor of Eukaryotes, and that high affinity is maintained in most extant animal phyla. However, affinity is low in nematodes and insects, raising questions about the physiological function of the interaction in species from these animal groups. Our findings demonstrate how an apparently established protein–protein interaction involved in cellular scaffolding in bilaterians can suddenly be subject to dynamic evolution including possible loss of function.

## Introduction

**Fig. 1.**
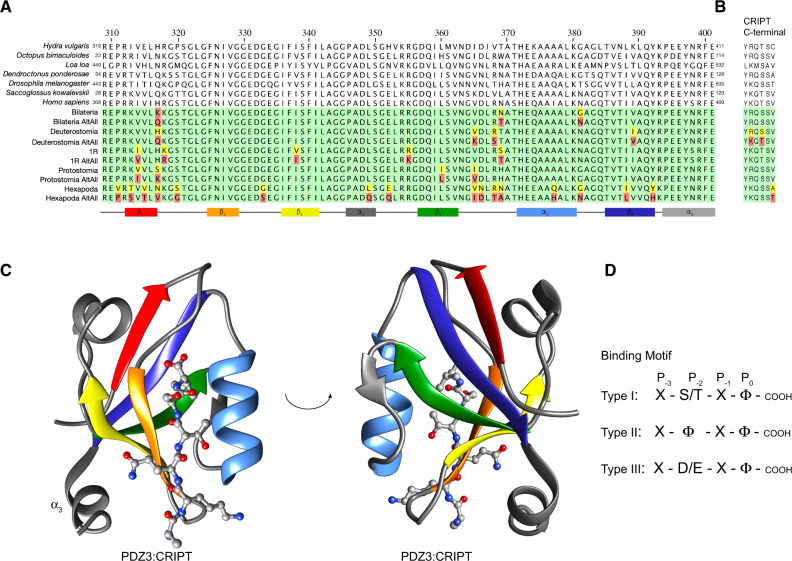
Extant and reconstructed ancient DLG PDZ3 and CRIPT sequences. (*A*) Alignment of DLG PDZ3 sequences. The sequence numbering of extant PDZ3 domains refers to the full-length DLG protein from the respective species. For simplicity, we use numbering of PDZ3 according to the human DLG4 sequence throughout the article. In the alignment of ancestral sequences (green background), residues with posterior probabilities <0.5 (red) and >0.5 but <0.8 (yellow) are highlighted. The secondary structure is from the crystal structure of DLG4 PDZ3 depicted in panel (*C*) with the corresponding color code. (*B*) Alignment of C-terminal CRIPT sequences. (*C*) Crystal structure of human DLG4 PDZ3 in complex with a CRIPT peptide (YKQTSV) (PDB ID: 5HEB). Secondary structure elements are marked in accordance with the alignment in panel (*A*). The structure was visualized using UCSF Chimera software. (*D*) Illustration of three common C-terminal PDZ-binding motif types. X, any amino acid residue; Φ, hydrophobic residue. Previous studies have shown that human DLG4 PDZ3 prefers a type I motif with a Val at position P_0_.

The postsynaptic density extends across the postsynaptic dendritic spine and is composed of receptors, signaling enzymes, cytoskeletal structural elements, and cytoplasmic scaffolding proteins. A major role of the postsynaptic density is to stabilize and anchor glutamate receptors such as AMPA and NMDA. Proteins from the discs large (DLG) family such as postsynaptic density protein-95 (PSD-95), also called DLG4, are involved in different protein–protein interactions in the postsynaptic density. Here, they control molecular organization and regulate synaptic strength by altering the function and distribution of AMPA receptors at the synapse (Chen et al. 2011). DLG4 contains five folded domains: Postsynaptic density-95/discs large/Zonula occludens (PDZ)1, PDZ2, PDZ3, Src homology 3 (SH3), and guanylate kinase like (GK). DLG4 is one of four paralogs in vertebrates together with SAP97 (DLG1), PSD-93 (DLG2), and SAP102 (DLG3), respectively. These four proteins arose as a result of two consecutive whole-genome duplications in the vertebrate lineage ∼440 Ma (McLysaght et al. 2002; Putnam et al. 2008). The amino acid sequences of the three PDZ domains found in each of these proteins are well conserved. In fact, the identity and similarity are high also for PDZ domains in the corresponding orthologous proteins in evolutionarily distantly related animals such as *Drosophila melanogaster* and the fresh-water polyp *Hydra vulgaris* (fig. 1*A*).

The PDZ domain fold is ancient and based on sequence similarity it can be traced in animals as well as in fungi and plants (Emes and Grant 2011), which contain a permuted variant (Ivarsson et al. 2008). Within the animal kingdom, there are examples of genomes containing hundreds of distinct PDZ domains, showing that its function as a protein–protein interaction module has been under positive selection following gene or genome duplications. This abundance of PDZ domains in the animal proteome is likely facilitated by its structural architecture allowing easy integration into other proteins (Harris and Lim 2001). PDZ domains consist of 80–100 residues and have a compact globular fold usually containing five to six antiparallel β strands and two α helices. However, DLG PDZ3 contains a third α helix at the C-terminus (α_3_) (fig. 1*C*).

Protein ligands of PDZ domains usually bind via their respective intrinsically disordered C-terminus to a binding groove in the PDZ domain. This groove is shaped by a conserved motif in the β_1_β_2_ loop (GLGF or variants of it), the β_2_ strand and the α_2_ helix. In most cases, the C-terminus of the protein ligand arranges as an antiparallel β strand in the groove together with the β_2_ strand (fig. 1*C*). The part of the protein ligand that binds to the PDZ domain is called the PDZ-binding motif (PBM). Affinity is primarily dictated by the protein ligand residues denoted P_−2_ and P_0_, where P_0_ is the C-terminal residue and P_−2_ the third residue from the C-terminus (fig. 1*D*). Early studies (Songyang et al. 1997; Stricker et al. 1997) led to classification of PBMs as type I (T/S-X-ϕ-COOH), type II (ϕ-X-ϕ-COOH), or type III (D/E-X-ϕ-COOH), based on the nature of the residues at P_−2_ and P_0_. However, the classification of PDZ domains only depending on two residues of the binding partner is an over simplification. Not only is the specificity of PDZ–ligand interactions usually dependent on more than P_−2_ and P_0_ residues of the ligand (Ernst et al. 2014) but also several properties of the PDZ domains themselves modulate binding, for example, residues outside of the binding groove (Ye et al. 2018) and extensions to the canonical PDZ domain such as α_3_ in DLG PDZ3 (Zeng et al. 2016).

One protein ligand for DLG PDZ3 is cysteine-rich interactor of PDZ3 (CRIPT) (Niethammer et al. 1998). This is an interaction that is well studied both from a structural and biophysical point of view, since it was the first structure solved of a PDZ domain with peptide ligand (Doyle et al. 1996). Full affinity of CRIPT (*K*_d_ in the low µM range for the *Homo sapiens* CRIPT:DLG4 PDZ3 interaction) is obtained with the six last amino acid residues (Saro et al. 2007; Gianni et al. 2011; Toto et al. 2016). CRIPT has a neurological function and is required for DLG to promote dendrite growth by AMPA activation (Beique et al. 2006; Zhang et al. 2008, 2017). However, certain animals including the model organisms *Dr. melanogaster* (where DLG was first identified) and *Caenorhabditis elegans* have CRIPT proteins that lack the classical PBM (fig. 1*B* and supplementary fig. 1, Supplementary Material online). In *Dr. melanogaster*, DLG is involved in a range of processes, including synaptic clustering of Shaker potassium channels (Tejedor et al. 1997), junction structure, cell polarity, and localization of membrane proteins. Recent work shows that CRIPT clusters next to DLG in the synapse, thereby promoting dendrite growth (Zhang et al. 2017). Furthermore, it was reported from knock down experiments that CRIPT is essential for DLG dependent dendrite growth (Zhang et al. 2017). Thus, there is some ambiguity whether CRIPT from, for example, *Dr. melanogaster*, binds PDZ3 domains when it lacks the classical PBM and whether the biological action of DLG and CRIPT is partially independent of the PDZ3:CRIPT interaction (Zhang et al. 2017).

**Fig. 2.**
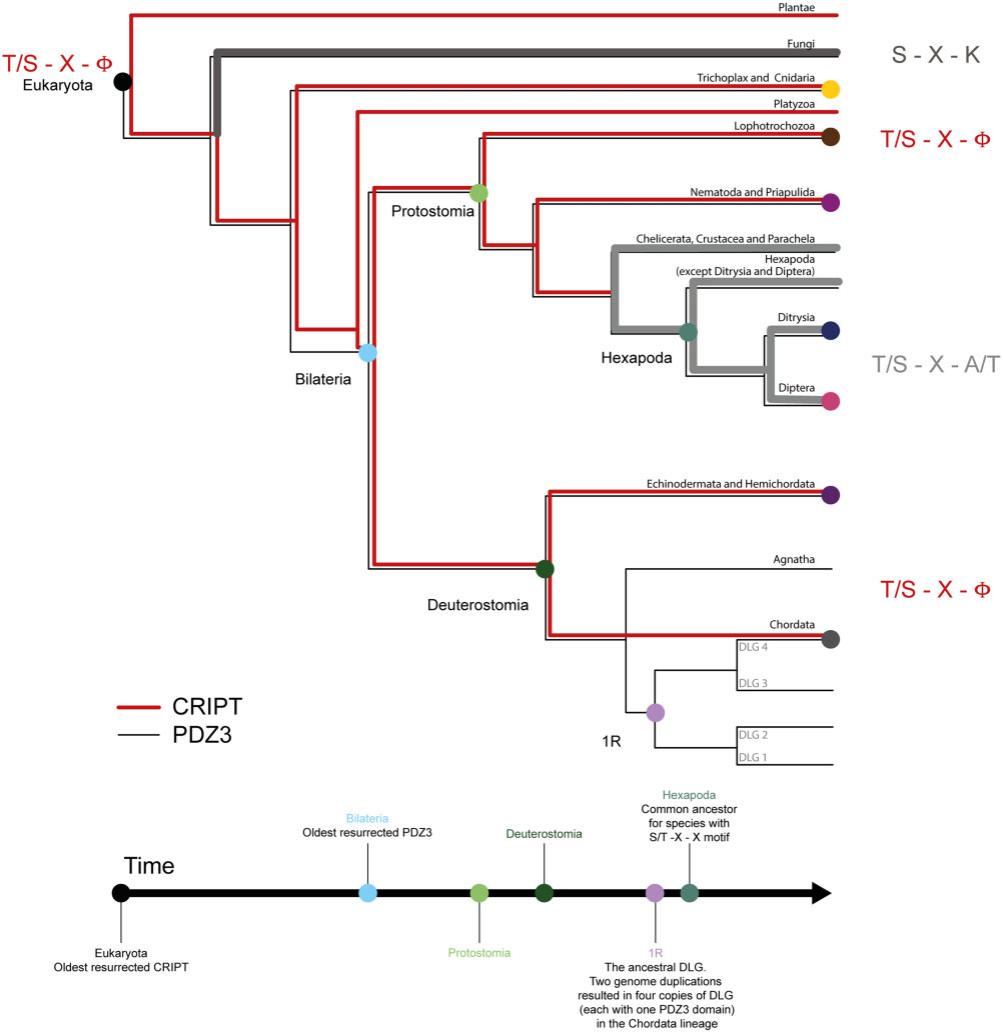
Evolution of DLG PDZ3 and the PDZ-binding motif in CRIPT. Simplified tree of organismal groups used for ancestral sequence reconstructions of CRIPT and PDZ3. The nodes in the phylogenetic tree are highlighted in the color code used throughout the article for extant and reconstructed ancestral CRIPT and PDZ3 domain variants. Evolution of CRIPT C-terminal-binding motif is highlighted by red (type I: T/S–X–Φ) to gray (undefined motif). Ancestral sequence reconstructions of CRIPT and PDZ3, respectively, were performed based on multiple sequence alignments and a species tree (CommonTree and OpenTree). In the reconstruction, 309 unique sequences of CRIPT from 498 different species and 249 unique sequences of PDZ3 from the DLG protein family from 324 different species were included.

Evolutionary biochemistry, that is, the combination of phylogenetic reconstruction of ancestral sequences with expression and experimental characterization of the ancient proteins with different methods is a powerful approach for understanding protein function (Hochberg and Thornton 2017), including protein–protein interactions (Hultqvist et al. 2017; Jemth et al. 2018; Wheeler et al. 2018). To better understand the evolution and hence function of the PDZ3:CRIPT interaction, we reconstructed sequences of ancestral phylogenetic tree-matched variants of PDZ3 and CRIPT and compared their binding affinity and stability to PDZ3 and CRIPT from seven extant species (fig. 2). We show that the PDZ3:CRIPT interaction has evolved such that the affinity has been maintained or slightly increased in most extant animal phyla (chordates, hemichordates, mollusks, and cnidarians), whereas affinity seems to be lost among most nematodes and insects. Our findings raise two questions: Is CRIPT the natural ligand for DLG PDZ3, and is control of AMPA and NMDA receptors independent of the PDZ3:CRIPT interaction, at least in nematodes and insects?

## Results

### Reconstruction of Ancestral Sequences

Protein sequences for DLG family members (including DLG4 and its vertebrate paralogs DLG1, DLG2, and DLG3) and CRIPT proteins were recovered from the NCBI database (NCBI Resource Coordinators) (NCBI Resource Coordinators). The final set of sequences for ancestral reconstruction consisted of 309 unique sequences of CRIPT from 498 different species and 249 unique sequences of DLG family PDZ3 domains from 324 different species (supplementary figs. 1 and 2, Supplementary Material online). The number of different species exceeded the number of unique sequences due to 100% sequence identity between several closely related species. Although all of the PDZ3 sequences were from Metazoan (animal) species, CRIPT sequences also included other kingdoms since homologous proteins with high sequence similarity were found in both fungi and plants. Within the animal kingdom, the similarity between homologous sequences of both DLG PDZ3 and CRIPT was very high (supplementary figs. 1 and 2, Supplementary Material online). Availability and quality of sequences were variable so the widest most representative data set was constructed using search methods, sequence alignment, filtering, and sorting using Python programing and manual curation. A good multiple sequence alignment is key for a reliable ancestral sequence reconstruction (Vialle et al. 2018). The high sequence identity and similarity for both PDZ3 (86.2% and 93.6%, respectively) and CRIPT (82% and 73%, respectively, for the C-terminal six residues) and, importantly, absence of insertions and deletions provided a high-quality alignment. Moreover, the high sequence similarity yielded high posterior probabilities (>0.8) for the maximum likelihood estimate (ML) of reconstructed ancestral sequences for most reconstructed positions: 94–97% in PDZ3 (except Hexapoda ancestor, 83%) and five or six out of six residues in the PBM of CRIPT (except Deuterostomia CRIPT, four out of six) (supplementary data excel files 1 and 2, Supplementary Material online). We were able to reconstruct PDZ3 from five historical time points, namely from the common ancestors of all extant bilaterians, deuterostomes, protostomes, and hexapods (insects), respectively, as well as from the common ancestor of all extant vertebrates containing four DLG paralogs (figs. 1*A* and 2). The PDZ3 from the latter ancestor is denoted 1R PDZ3, since the four DLG paralogs arose as a result of two consecutive whole-genome duplications called 1R and 2R, respectively (McLysaght et al. 2002; Putnam et al. 2008). The C-terminus, that is, the PBM of CRIPT was reconstructed at six historical time points, namely from the common ancestors of all extant eukaryotes, bilaterians, deuterostomes, protostomes, vertebrates (1R), and hexapods, respectively (figs. 1*B* and 2 and supplementary fig. 3, Supplementary Material online). In addition to the ancient ones, we expressed and characterized DLG PDZ3 and CRIPT variants from the following extant species representing different distantly related animal groups: The nonbilaterian animal *Hy. vulgaris* (a cnidarian), *Octopus bimaculoides* (California two-spot octopus, a mollusk, and protostome), *Loa loa* (eye worm—a nematode or roundworm causing the disease loiasis, protostome), *Dendroctonus ponderosae* (mountain pine beetle, an insect, protostome), *Dr. melanogaster* (fruit fly, insect, protostome), *Saccoglossus kowalevskii* (acorn worm, a hemichordate, deuterostome), and *Ho. sapiens* (representing chordates, deuterostome) (figs. 1*A* and *B* and 2 and supplementary fig. 3, Supplementary Material online). We use “native interaction” to denote interactions between tree-matched or species-matched PDZ3:CRIPT interactions. Based on sequence alignment and structure prediction, it appears that all ancestral and extant DLG PDZ3 domains have a similar structure with the usual PDZ fold consisting of six β strands and two α helices, but also including a third C-terminal α helix (α_3_), present in the crystal structures of human DLG4 PDZ3 (Doyle et al. 1996) (supplementary fig. 4, Supplementary Material online). During revision, we received comments on our preprint that RAxML is not optimized for ancestral sequence reconstruction. We therefore repeated the reconstruction of both PDZ3 and CRIPT with two other software, RAxML-NG and PAML, respectively, and with two different trees (CommonTree and OpenTree). Overall, the results were consistent (supplementary data excel file 2, Supplementary Material online), but in particular one reconstructed PDZ3 variant (the deuterostome ancestor) differed from that predicted by RAxML. The new ML variant predicted by PAML was therefore expressed, purified, and subjected to the same experiments as the other PDZ3 variants described below. The PAML variant of deuterostome PDZ3 was found to exhibit close to identical properties as that predicted by RAxML (supplementary fig. 5, Supplementary Material online).

### Evolution of PDZ3:CRIPT Interaction

From the sequence alignment and phylogeny of the C-terminal residues in CRIPT (supplementary fig. 1, Supplementary Material online), we can follow the evolution of the PBM (fig. 2 and supplementary fig. 3, Supplementary Material online). Among viridiplantae, plants and mosses show a striking and complete conservation of a type 1 motif (YKQSNV), whereas green algae have a polar residue at position P_0_. The majority of animal phyla including vertebrates, annelids, and mollusks (except Euthyneura, i.e., snails and slugs) have CRIPT with a classical type 1 PBM. However, fungi and arthropods (including insects, spiders, and crustaceans) appear to lack a PBM, although there are a few exceptions. Interestingly, CRIPT from the cnidarian *Hy. vulgaris* ends with a Cys (fig. 1*B*).

In accordance with the alignment and phylogeny, CRIPT from the last common ancestor of all extant Eukaryotes probably contained a classical type 1 PBM (YKQSSV) (fig. 2 and supplementary fig. 3, Supplementary Material online). A type 1 PBM was likely also present in the ancestor of all animals but has since mutated in distinct animal phyla and orders. Clearly, the ancestor of bilaterian animals contained a type 1 PBM and so did the ancestors of the two major bilaterian groups, protostomes and deuterostomes. However, the last common ancestor of today’s arthropods likely carried a mutated PBM in CRIPT, ending with Ala or Thr, instead of the canonical Val residue.

**Fig. 3.**
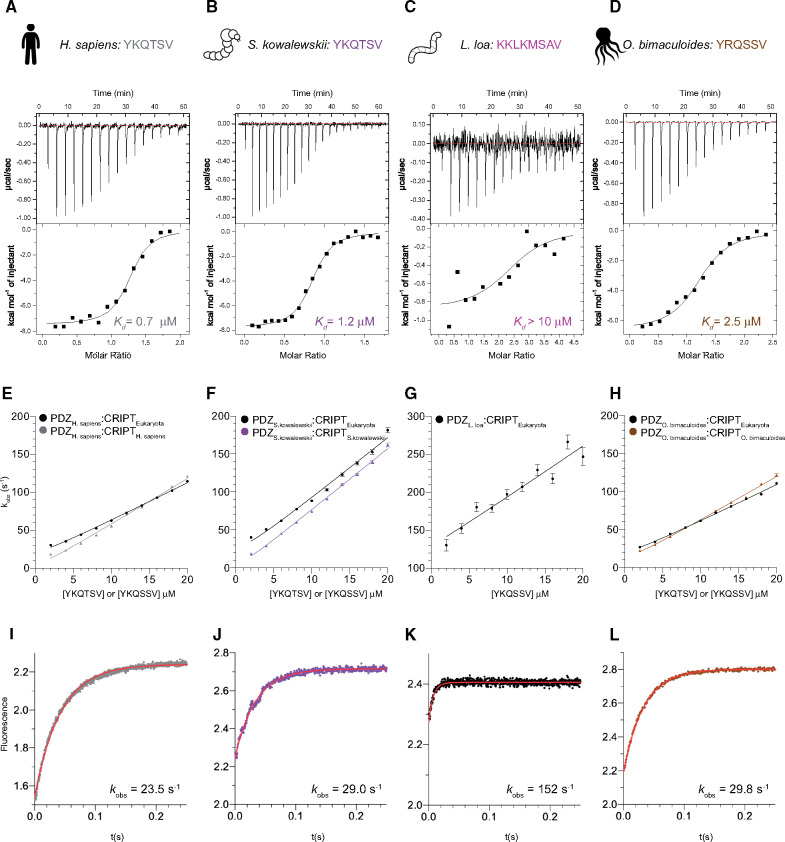
Characterization of the PDZ3:CRIPT interaction from extant species with a type 1 PBM in CRIPT. (*A–D*) Examples of ITC experiments for native PDZ3:CRIPT interactions for four extant species: *Homo sapiens*, *Saccoglossus kowalevskii*, *Loa loa*, and *Octopus bimaculoides*. (*E–L*) Binding kinetics from stopped-flow experiments for the same native interactions as in panel (*A–D*), and also including experiments with a reconstructed CRIPT from the ancestor of all eukaryotes (CRIPT_Eukaryota_). (*E–H*) Observed rate constants were obtained from kinetic-binding traces (examples shown in panels *I–L*) and plotted as function of CRIPT concentration at a constant concentration of PDZ3 (1 µM) to estimate the association rate constant *k*_on_. (*I–L*) Examples of kinetic-binding traces from stopped-flow experiments using 4 µM CRIPT and 1 µM PDZ3. The fitted line (red) is a single exponential from which the observed rate constant *k*_obs_ was obtained. *k*_off_ was determined in a separate experiment as explained in Materials and Methods section, and *K*_d_ calculated as *k*_off_/*k*_on_. Experiments were performed at 25 °C for ITC and at 10 °C for stopped-flow.

**Fig. 4.**
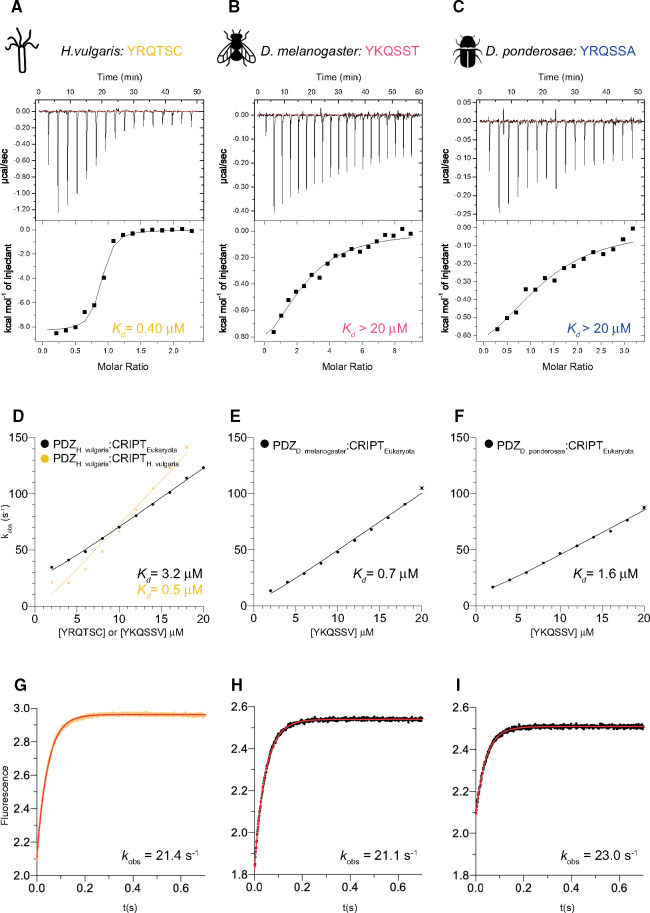
Characterization of the PDZ3:CRIPT interaction from extant species without a type 1 PBM in CRIPT. (*A–C*) Examples of ITC experiments for native PDZ3:CRIPT interactions for three extant species (*Hydra vulgaris*, *Drosophila melanogaster*, and *Dendroctonus ponderosae*, respectively). (*D–I*) Binding kinetics from stopped-flow experiments for the native *Hy. vulgaris* interaction and also experiments with the respective PDZ3 and a reconstructed CRIPT from the ancestor of all eukaryotes (CRIPT_Eukaryota_). (*D–F*) Observed rate constants were obtained from kinetic-binding traces (examples shown in panels *G–I*) and plotted as function of CRIPT concentration at a constant concentration of PDZ3 (1 µM) to estimate the association rate constant *k*_on_. (*G–I*) Examples of kinetic-binding traces from stopped-flow experiments using 4 µM CRIPT and 1 µM PDZ3. The fitted line (red) is a single exponential from which the observed rate constant *k*_obs_ was obtained. *k*_off_ was determined in a separate experiment as explained in Materials and Methods section, and *K*_d_ calculated as *k*_off_/*k*_on_. Experiments were performed at 25 °C for ITC and at 10 °C for stopped-flow.

One intriguing notion is that the C-terminal residues P_−1_ to P_−5_ are highly conserved even among CRIPTs with Ala or Thr at position P_0_. This raises the question whether CRIPTs without a canonical type 1 PBM still retain binding to their native DLG PDZ3? To address this question, PDZ3 and CRIPT from seven extant and five ancestral species were subjected to binding experiments using ITC and stopped-flow spectroscopy (figs. 3 and 4).

**Fig. 5.**
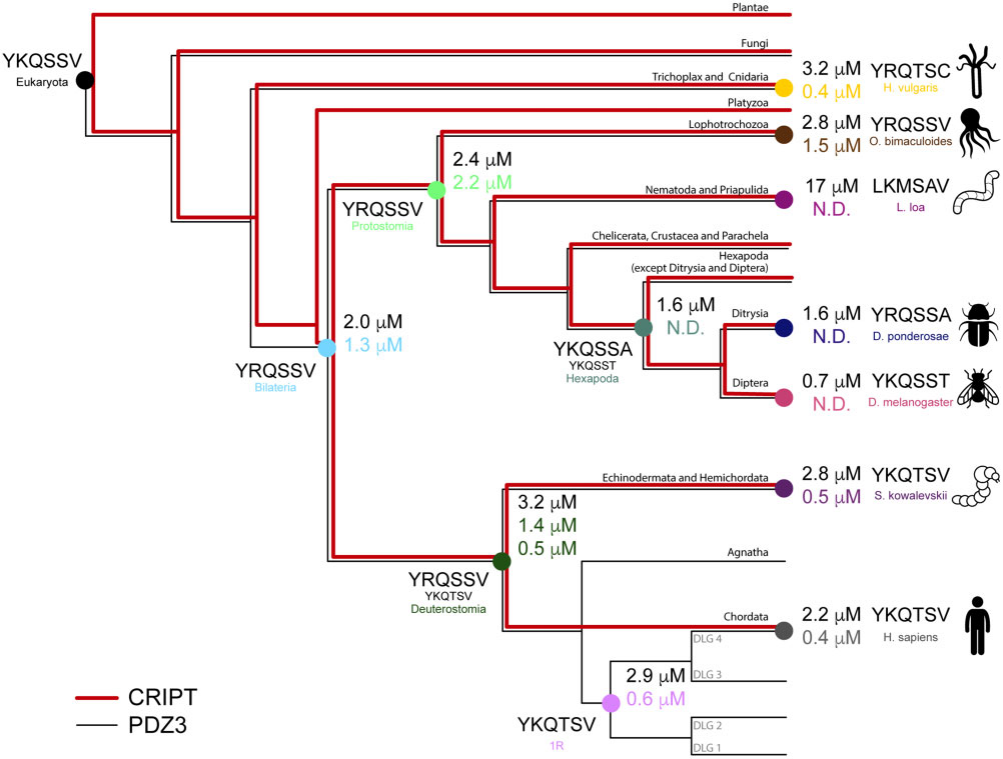
Evolution of affinity for the PDZ3:CRIPT interaction. Simplified species tree with nodes for selected ancestral and extant species used in the study. The native phylogenetic tree-matched affinity for each interaction is shown in color code text at each node and the affinity between each PDZ3 and Eukaryota CRIPT is shown in black text. For the native interaction in the deuterostome node, two CRIPT peptides were tested due to low posterior probability for Ser at position P_−2_. *K*_d_ values were determined by stopped-flow experiments at 10 °C. N.D., not determined because of too low affinity.

The binding of *Ho. sapiens* CRIPT to *Ho. sapiens* DLG4 PDZ3 is well characterized (Doyle et al. 1996; Niethammer et al. 1998; Saro et al. 2007; Gianni et al. 2011; Toto et al. 2016), and we report a similar binding affinity (0.40 µM) as previous studies (figs. 3 and 5; tables 1 and 2). Furthermore, PDZ3 from *Hy. vulgaris* (with a C-terminal Cys residue), *O. bimaculoides*, *S. kowalevskii*, and the common ancestors of bilaterian animals, protostomes, deuterostomes, and vertebrates (1R), respectively, were all found to bind to their tree-matched native CRIPT with low µM affinity (figs. 3–5;tables 1 and 2; and supplementary fig. 6, Supplementary Material online). On the other hand, PDZ3 domains from *Dr. melanogaster*, *De. ponderosae*, and the ancestor of hexapods bind poorly to their respective native CRIPT, which lacks a type 1 PBM. Although the interactions were too weak for stopped-flow spectroscopy, ITC experiments provided a rough estimate of the affinities (*K*_d_≥10–30 µM) (table 2;figs. 4 and 5). More surprisingly, PDZ3 from the nematode *L. loa* was also found to bind poorly to its native CRIPT even though it contains a type 1 PBM with a C-terminal Val (fig. 3).

**Table 1.** Binding Constants for Extant and Reconstructed Ancestral PDZ3:CRIPT Interactions from Kinetic Experiments.

CRIPT	PDZ3	*k*_on_ (µM^−1^ s^−1^)	*k*_off_ (s^−1^)	*K*_d_ (µM)
YKQSSV	*H. sapiens*	5.32±0.04	11.58±0.01	2.18±0.02
	1R	5.3±0.1	15.3±0.3	2.89±0.09
	Deuterostomia	7.4±0.2	23.61±0.03	3.17±0.07
	Hexapoda	7.3±0.1	11.5±0.1	1.58±0.03
	Protostomia	6.5±0.1	15.5±1.3	2.4±0.2
	Bilateria	5.9±0.1	11.9±0.3	2.04±0.06
	*S. kowalevskii*	7.6±0.2	20.8±0.3	2.75±0.07
	*D. melanogaster*	5.4±0.1	3.60±0.01	0.67±0.01
	*D. ponderosae*	4.3±0.1	6.94±0.03	1.63±0.03
	*H. vulgaris*	5.68±0.05	18.0±0.2	3.17±0.05
	*O. bimaculoides*	4.9±0.1	13.6±0.2	2.80±0.05
	*L. loa*	7.0±0.7	117±3	17±2
	*L. loa* P335A	8.4±0.2	70.6±1.0	8.5±0.3
	*L. loa* Q399R	8.2±0.1	38.0±0.1	4.66±0.07
	*L. loa* α_3_	8.8±0.1	14.4±1.0	1.64±0.12
YKQTSV	*H. sapiens*	6.3±0.1	2.49±0.01	0.395±0.005
	1R	6.2±0.1	3.46±0.02	0.56±0.01
	Deuterostomia	9.3±0.2	5.02±0.03	0.54±0.01
	*S. kowalevskii*	8.1±0.1	4.19±0.04	0.52±0.01
YRQSSV	Deuterostomia	9.4±0.2	13.4±0.1	1.43±0.03
	Protostomia	4.6±0.3	10.3±0.1	2.2±0.2
	Bilateria	5.1±0.2	6.8±0.1	1.33±0.06
	*O. bimaculoides*	5.8±0.1	8.5±0.1	1.47±0.03
	*H. sapiens*	5.5±0.1	6.85±0.02	1.24±0.03
YRQTSC	*H. vulgaris*	10.7±0.3	4.7±0.2	0.44±0.02
	*H. sapiens*	10.3±0.2	3.32±0.07	0.36±0.01

Note.—Rate and equilibrium constants were determined for the PDZ3:CRIPT interaction for extant and ancestral PDZ3 domains binding its respective native CRIPT (YKQTSV, YRQSSV, or YRQTSC). In addition, binding experiments were performed for all PDZ3 domains to the ancestral Eukaryota CRIPT (YKQSSV) and for all CRIPTs to *Homo sapiens* PDZ3. Native interactions are underlined. Stopped-flow experiments were used to determine the association rate constant (*k*_on_) from binding experiments and the dissociation rate constant (*k*_off_) from displacement experiments. *K*_d_ values were calculated from the ratio of *k*_off_ and *k*_on_*.* All data were obtained at 10 °C in 50 mM sodium phosphate, pH 7.45, 21 mM KCl (*I* = 150).

**Table 2.** Binding Constants for Extant and Reconstructed Ancestral PDZ3:CRIPT Interactions Monitored by Isothermal Titration Calorimetry (ITC).

CRIPT	PDZ3	Δ*H* (kcal mol^−1^)	*T*Δ*S* (kcal mol^−1^)	*K*_d_ (µM)
YRQSSA	*D. ponderosae*	−0.8±0.2	5.4	31±11
	Hexapoda	−2.3±2.0	3.5	56±25
GKKMMSTKNYRQSSA	*D. ponderosae* α_3_	−1.0±0.02	5.5	15±4
YRQSST	*D. melanogaster*	−1.0±0.2	5.3	27±20
	Hexapoda	−1.1±0.1	5.3	21±4
YRQSpST	*D. melanogaster* α_3_	−1.6±0.01	4.4	40±9
GKKIMNTKNYKQSST	*D. melanogaster* α_3_	−0.5±0.02	5.6	34±13
KKLKMSAV	*L. loa*	−1.1±0.2	5.7	12±7
GKKMADTKKLKMSAV	*L. loa* α_3_	−0.8±0.02	6.3	6.8±2
YKQTSV	*H. sapiens*	−7.5±0.2	0.9	0.7±0.1
	1R	−7.6±0.2	1.1	0.5±0.1
	Deuterostomia	−8.8±0.1	−0.6	0.9±0.1^a^
	*S. kowalevskii*	−7.8±0.1	0.3	1.2±0.1
YRQSSV	Deuterostomia	−6.8±0.1	0.9	2.1±0.2
	Protostomia	−6.4±0.2	1.3	2.4±0.3
	Bilateria	−6.8±0.2	1.1	1.6±0.3
	*O. bimaculoides*	−6.6±0.2	1.0	2.5±0.4
YRQTSC	*H. vulgaris*	−8.3±0.2	0.5	0.4±0.1

Note.—Thermodynamic parameters were determined at 25 °C in 50 mM sodium phosphate, pH 7.45, 21 mM KCl (*I* = 150). A longer *Loa loa* CRIPT peptide was used (eight residues) due to low solubility of the hexamer.^a^AltAll interaction for the Deuterostomia PDZ3:CRIPT interaction.

To directly compare each PDZ3 domain with regard to binding of CRIPT with a type 1 PBM, the affinity of CRIPT from the ancestor of all eukaryotes (YKQSSV) was measured with all PDZ3 domains included in the study. The ancestral Eukaryota CRIPT was found to bind to all resurrected and present-day PDZ3 domains, including insect PDZ3 domains, in the low µM range (figs. 3–5;table 1; and supplementary fig. 6, Supplementary Material online). However, the binding of Eukaryota CRIPT to the nematode *L. loa* PDZ3 was somewhat weaker than for other PDZ3 domains (17 µM), suggesting that *L. loa* PDZ3 has a binding groove with a slightly different character than PDZ3 domains from the other investigated extant and ancestral species. Thus, although nematodes, the sister phylum of arthropods, has retained the type 1 PBM in CRIPT the high affinity of the PDZ3:CRIPT interaction is lost, suggesting that it may not be functional in either group of animals. Another option is that the PDZ3:CRIPT interaction is functional but interactions outside the canonical groove are essential for full affinity among Ecdysozoa as observed for some other PDZ:ligand complexes (Pan et al. 2011; Li et al. 2014; Zeng et al. 2016; Ye et al. 2018) or that the PDZ3:CRIPT interaction is posttranslationally regulated (Sundell et al. 2018). Obviously, lower affinity could have evolved as a response to a certain course of events leading to for example high local cellular concentrations of DLG and CRIPT. Finally, a trivial explanation is that the PDZ3 domains used in the experiments were not properly folded. Although it is challenging to test intracellular expression levels, we attempted to partially address the other issues.

### Mutational Analysis to Resolve the Low CRIPT Affinity of PDZ3 from *L. loa*

The *L. loa* PDZ3:CRIPT interaction is particularly interesting since it appears as if the PDZ3 domain has lost affinity for CRIPT with type 1 PBM. Sequence alignment (fig. 1*A*) and structural prediction of the PDZ3 variants show that *L. loa* PDZ3 differs in several amino acid positions as compared with other PDZ3 domains, which inspired us to check whether interactions outside the canonical groove could be essential for the PDZ3:CRIPT interaction. *Loa loa* PDZ3 has several unique residues at positions that previously have been reported to be important for binding affinity (supplementary fig. 7, Supplementary Material online), for example, Gly→Pro at position 335 in the β_2_β_3_ loop next to the canonical-binding groove and Arg→Gln at position 399 in α_3_. The presence of Pro335 and/or Gln399 is rare; they are only found in 12 sequences among the 253 sequences used for the ancestral sequence reconstruction. Furthermore, a previous study showed an increased β_2_β_3_ loop flexibility if the salt-bridge between Arg 399 in α_3_ and Glu 334 in the β_2_β_3_ loop in *Ho. sapiens* DLG4 PDZ3 is missing (Mostarda et al. 2012). The interaction is very stable and formed both in apo- and in peptide-bound states. Thus, two single point mutations, P335G and Q399R, were introduced into *L. loa* PDZ3, to test if the amino acid substitutions can explain the weak binding to CRIPT. Indeed, the affinity increased 2- and 3-fold, respectively, toward ancestral Eukaryota CRIPT (table 1), supporting the notion that the derived substitutions in *L. loa* PDZ3 indeed weakened the interaction with CRIPT.

### Global Stability of Ancestral and Extant PDZ3 Domains

**Fig. 6.**
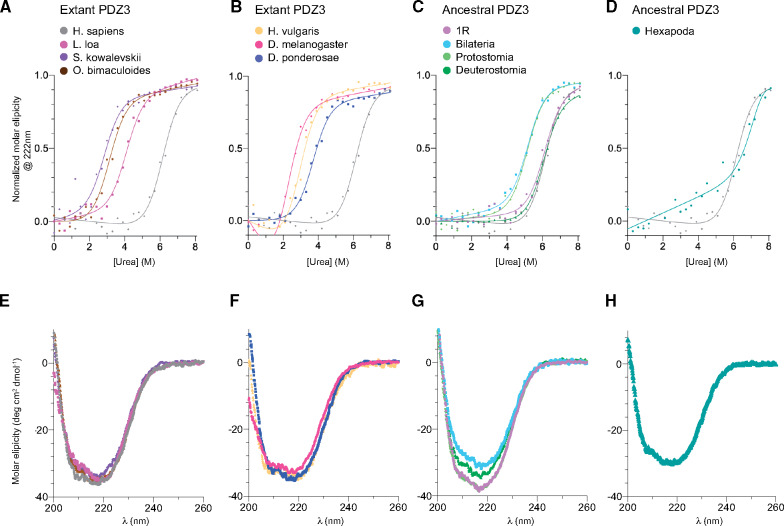
Secondary structure and global stability of extant and resurrected ancestral PDZ3 domains. (*A–D*) Urea denaturation (0–8.1 M) of extant and ancestral PDZ3 variants, as monitored by circular dichroism at 222 nm. Data were fitted to a two-state model for protein (un)folding. (See table 3 for fitted parameters.) To facilitate comparison, the urea denaturation curve of *Homo sapiens* PDZ3 (gray) is present in all graphs. (*E–H*) Secondary structure content analyzed by circular dichroism between 200 and 260 nm. Each spectrum is an average of five individual scans measured at 10 °C in 50 mM sodium phosphate, pH 7.45, 21 mM KCl (*I*=150 mM).

Because of the high general interest in evolution of stability (Williams et al. 2006; Gaucher et al. 2008; Risso et al. 2014; Trudeau et al. 2016; Wheeler et al. 2016; Sternke et al. 2019), we addressed this question using our reconstructed PDZ3 variants. Thus, we determined the thermodynamic stability of extant and resurrected ancient PDZ3 variants using urea denaturation experiments monitored by far UV circular dichroism, which detects secondary structure and is a robust probe of global folding (fig. 6*A*–*D*, table 3, and supplementary fig. 8*F*–*J*, Supplementary Material online). Thermodynamic stability generally correlates with the thermal midpoint of unfolding, which could not be well determined due to precipitation. All PDZ3 domains in the study unfolded by an apparent two-state mechanism in equilibrium urea denaturation experiments. However, because of uncertainties in curve fitting, as described below, we were careful in the interpretation regarding how the thermodynamic stability has changed during evolution. All resurrected PDZ3 domains (without an extended α_3_ helix) were found to be more stable in comparison to extant PDZ3 domains, except *Ho. sapiens* PDZ3. Generally, we observed a slight increase in the urea midpoint for the deuterostome lineage from bilaterian to *Ho. sapiens* DLG4 PDZ3. However, there is some ambiguity in the actual stability of the PDZ3 variants due to uncertainty in determination of the *m*_D-N_ value, which reflects the change in solvent-accessible hydrophobic surface area upon denaturation and which is directly related to the stability, that is, the difference in Gibbs free energy between the denatured and the native state: *ΔG*_D-N_=[Urea]_50%_ × *m*_D-N_. It is fair to assume that all PDZ3 variants of similar sequence length also have a similar *m*_D-N_ value. Therefore, we fitted the data with a shared *m*_D-N_ value, which gives a more robust estimate of the differences in *ΔG*_D-N_ between PDZ3 variants. In addition, some PDZ3 variants did not show complete unfolding at the highest urea concentration, resulting in a lower accuracy of the fitted parameters. Nevertheless, it is clear that the extant and ancient PDZ3 domains included in the study display unfolding free energies spanning from 2.6 to 7.6 kcal mol^−1^ (Hexapoda ancestor likely higher). This wide range suggests that for this particular PDZ domain there is no overall evolutionary trend regarding stability, in the time window from the most recent common ancestor of bilaterian animals (∼550–600 My) until today. However, in specific lineages, stability has been maintained (human, 7.6 kcal mol^−1^), whereas in other lineages such as the one leading to *Dr. melanogaster*, stability has decreased (2.6 kcal mol^−1^).

**Table 3.** Global Stability of PDZ3 Domains.

PDZ3	[Urea]_50%_^a^ (M)	Δ*G*_D-N_^a^ (kcal mol^−1^)	[Urea]_50%_^b^ (M)	Δ*G*_D-N_^b^ (kcal mol^−1^)	*m*_D-N_ ^b^ (kcal mol^−1^ M^−1^)
*H. sapiens*	6.2±0.4	7.6±1.3	6.5±0.4	6.3±1.3	1.0±0.2
*S. kowalevskii*	2.7±0.1	3.3±0.9	2.7±0.1	4.3±0.9	1.6±0.3
*D. melanogaster*	2.1±0.1	2.6±0.3	2.1±0.1	2.4±0.3	1.2±0.1
*D. melanogaster* α_3_	3.0±0.1	4.0±0.4^c^	3.0±0.04	4.4±0.3	1.5±0.1
*D. ponderosae*	3.7±0.1	4.5±0.8	3.7±0.1	5.1±0.8	1.4±0.2
D. ponderosae α_3_	4.5±0.1	6.0±0.6^c^	4.4±0.05	7.2±0.7	1.6±0.2
*L. loa*	4.1±0.1	5.0±1.4	4.0±0.1	7.0±1.4	1.7±0.3
*L. loa* α_3_	5.7±0.1	7.7±0.7^c^	5.8±0.2	6.6±1.4	1.1±0.2
*O. bimaculoides*	3.1±0.1	3.8±0.8	3.2±0.1	4.6±0.8	1.5±0.3
*H. vulgaris*	2.9±0.1	3.6±0.8	2.9±0.1	3.9±0.8	1.3±0.3
Bilateria	5.2±0.2	6.4±0.6	5.4±0.2	4.1±0.6	0.8±0.1
Bilateria AltAll	5.75±0.03	7.1±0.3	5.78±0.04	6.8±0.3	1.17±0.05
Protostomia	5.1±0.1	6.3±1.2	5.1±0.1	6.4±1.2	1.2±0.2
Protostomia AltAll	5.3±0.2	6.5±1.4	5.3±0.2	5.9±1.4	1.1±0.3
Deuterostomia	6.0±0.1	7.4±0.6	6.1±0.1	7.1±0.6	1.2±0.1
Deuterostomia AltAll	4.7±0.1	5.8±0.9	4.7±0.1	5.1±0.9	1.1±0.2
Hexapoda	7.0±0.7	10±4.0^d^	6.7±0.4	17±13	2.5±2.0
Hexapoda AltAll	5.2±0.3	7.4±2.8^d^	5.3±0.4	5.8±3.0	1.1±0.5
1R	6.1±0.2	7.5±2.4	5.9±0.2	9.4±2.3	1.6±0.4
1R AltAll		^e^	6.6±0.5	13±7.2	1.9±1.1

Note.—Global stability of PDZ3 domains was determined by urea denaturation experiments monitored by circular dichroism at 222 nm (see fig. 6). Experimental data were fitted to a two-state model for denaturation to obtain the concentration of urea, where 50% of the protein is denatured ([Urea]_50%_), the cooperativity of the unfolding (*m*_D-N_ value) and the stability (Δ*G*_D-N_) as the product of [Urea]_50%_.and *m*_D-N_.

a The *m*_D-N_ value was shared among the data sets in the curve fitting; *m*_D-N_=1.23 kcal mol^−1^ M^−1^.

b Free fitting of both [Urea]_50%_ and *m*_D-N_.

Exceptions: ^c^α_3_ PDZ3 *m*_D-N_=1.35 kcal mol^−1^ M^−1^; ^d^Hexapoda and Hexapoda AltAll *m*_D-N_=1.43 kcal mol^−1^ M^−1^; and ^e^1R AltAll.

### The α_3_ Extension Increases the Stability of PDZ3

**Fig. 7.**
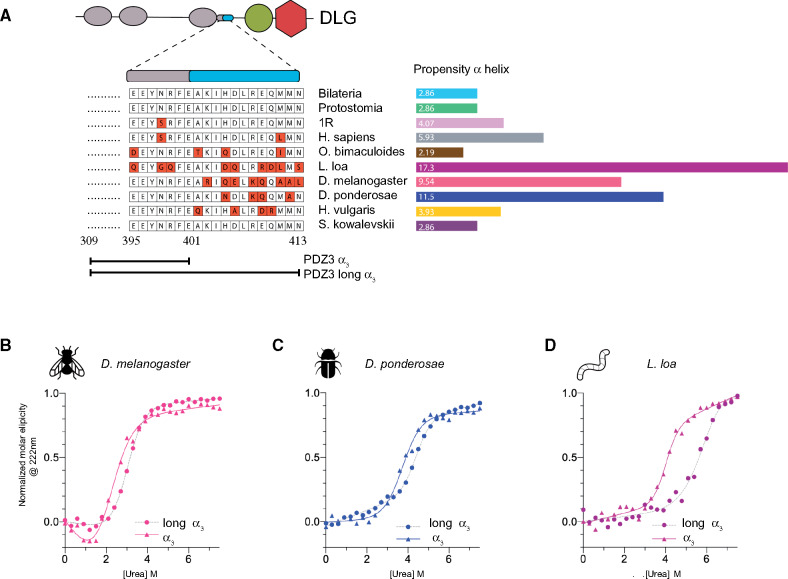
Properties of PDZ3 domains with an extended α_3_. (*A*) Schematic illustration of DLG4 with the PDZ1, PDZ2, PDZ3, SH3, and GK domains together with a sequence alignment of the extended α_3_ helix (19 aa) for the PDZ3 variants from extant and ancestral species included in the study. Alpha helix propensity was calculated by the AGADIR software at 283K, *I*=150 mM, pH 7.45. (*B–D*) Urea denaturation experiments of PDZ3 from *Drosophila melanogaster*, *Loa loa*, and *Dendroctonus ponderosae*, respectively, with and without the α_3_ extension, as monitored by circular dichroism at 222 nm. (See table 3 for fitted parameters.)

Most studies of DLG4 PDZ3 have been performed on a construct based on the original crystal structure (Doyle et al. 1996). However, in DLG4, PDZ3 is part of the supramodule PDZ3–SH3–GK in which an extended α helix (α_3_) connects PDZ3 and SH3 (fig. 7*A*), but only 7 of the 19 amino acid residues of the linker are present in the constructs investigated in the present work and in previous publications. Recent studies have reported that the entire α_3_ and a peptide ligand longer than six residues are required for specific PDZ:ligand interactions (Zeng et al. 2016, 2018; Ye et al. 2018). The α_3_ extension does not significantly affect the affinity between *Ho. sapiens* PDZ3–SH3–GK and a 6-mer or 15-mer CRIPT peptide (Laursen et al. 2020). However, as compared with the ancestral bilaterian PDZ3, the α_3_ region is highly conserved in *Ho. sapiens* PDZ3 with only two substitutions, whereas *De. ponderosae*, *Dr. melanogaster*, and *L. loa* PDZ3 have four, eight, and nine substitutions, respectively, in the 19 residue long primary structure of α_3_ (fig. 7*A*). We calculated the α helix propensity of isolated α_3_ (residues 395–413) for all PDZ3 variants in the present study using the AGADIR software (Munoz and Serrano 1994). The helical propensity varied from 2% to 17% (fig. 7*A*), suggesting that the stability of α_3_ may have changed during evolution. (The helical propensity refers to an isolated peptide but it could correlate with stability in the context of the folded protein.) Interestingly, *L. loa*, *Dr. melanogaster*, and *De. ponderosae* PDZ3 have the highest helix propensity in α_3_. Therefore, we decided to express, purify, and analyze PDZ3 from these three species with a full-length 19 residue α_3_ to assess the effect on binding and stability. We observed a slightly higher thermodynamic stability for the longer variants of PDZ3 in comparison to PDZ3 domains without an extended α_3_ such that they approach the stability of human PDZ3 (fig. 7*B–D* and table 3).

Like the PDZ3 variants without α_3_ extension, the affinities between the extended α_3_ PDZ3 variants from *L. loa*, *Dr. melanogaster*, and *De. ponderosae* and their respective native CRIPT ligands (15-mer) were too low to be measured by stopped-flow spectroscopy. But, with ITC, it was again possible to obtain a rough estimate of the *K*_d_ values. The affinity between *Dr. melanogaster* PDZ3 and CRIPT was not affected by either the α_3_ extension or a longer CRIPT peptide (table 2). Similarly, although the affinity seems to increase 2-fold for the respective native *De. ponderosae* and *L. loa* PDZ3:CRIPT interactions, this effect is within error of the experiment (table 2). However, the α_3_ extension increased the affinity of *L. loa* PDZ3 for the Eukaryota CRIPT significantly, by 10-fold (table 1), suggesting that the α_3_ extension of PDZ3, perhaps even in the context of a supramodule, needs to be considered if a weak interaction is analyzed.

### Negative Regulation by Phosphorylation

Reversible phosphorylation results in positive or negative regulation of protein–protein interactions and therefore plays an important role for cellular signaling. Protein phosphorylation mainly occurs at Ser, Thr, and Tyr residues. Thus, with regard to the PDZ3:CRIPT interaction, phosphorylation at P_−2_ (Thr) in CRIPT disables binding to PDZ3 (Sundell et al. 2018). However, *Dr. melanogaster* CRIPT lacks the type 1 motif but has three possible phosphorylation sites, ending with SST. Therefore, to rule out that the weak interaction between *Dr. melanogaster* PDZ3 and its native CRIPT could be rescued by phosphorylation of CRIPT, we tested phosphorylation at all three potential sites at the C-terminus of CRIPT. However, phosphorylation of *Dr. melanogaster* CRIPT at Ser_−1_ did not affect the binding (table 2), whereas phosphorylation of Ser_−2_ and Thr_0_ seems to weaken the affinity further since no binding could be detected by either stopped-flow or ITC. Thus, if anything, the PDZ3:CRIPT interaction is negatively regulated by phosphorylation in *Dr. melanogaster*.

### The Conclusions Based on Resurrected Maximum Likelihood Variants of PDZ3 Are Robust to Sequence Uncertainty

Finally, our historical description of the PDZ3:CRIPT evolution is contingent on accurate native affinities and stabilities for the resurrected proteins. Thus, even if the reconstructed sequences are not correct, what about the experimentally determined properties of the proteins? Different approaches can be applied to check the robustness of results based on the ML variant, for example generating a library of variants of the ML ancestral sequence by point mutations (Yokoyama and Radlwimmer 2001) or using Bayesian sampling to reconstruct a set of sequences by sampling amino acids over the posterior probability distribution (Yang and Rannala 1997). However, an easier approach is to reconstruct a “worst-case scenario” variant. Here, one sequence is reconstructed in which every amino acid residue with a posterior probability below a certain threshold, for example, 0.8, is replaced by the residue with the second-highest probability at that position. Such a variant, denoted AltAll (alternative residue at all positions), represents a very unlikely worst-case scenario that can be used to assess how robust conclusions are to errors in the reconstructed ML sequences (Anderson et al. 2016b; Eick et al. 2017) simply by comparing the ML and AltAll proteins. If they display the same affinity for a ligand, that affinity is very likely to be the true ancestral affinity (fig. 1*A* and supplementary figs. 3 and 6, Supplementary Material online).

Thus, we used the AltAll approach as a relatively conservative test of robustness with a single protein variant as control. Worst-case scenario AltAll sequences from CRIPT and PDZ3 were reconstructed, expressed, and subjected to the same experiments as the ML variants to validate the conclusions based on the resurrected ML proteins. The C-termini of ancestral CRIPT could be reconstructed with high confidence due to the strong conservation observed in extant species. Therefore, AltAll CRIPT variants were only tested in two cases, for the ancestor of hexapods and ancestor of deuterostomes, respectively, both of which had one ambiguous position (fig. 1*B* and supplementary figs. 3 and 6, Supplementary Material online). AltAll variants of PDZ3 contained three to five substitutions for 1R, Deuterostomia, Protostomia, and Bilateria PDZ3, at positions outside the canonical-binding groove (fig. 1*A*). The primary structure of AltAll for the ancestral Hexapoda PDZ3 contained 16 substitutions in comparison to the ML sequence. This is because PDZ3 at the Hexapoda node is resurrected from relatively few sequences and from species with a large number of amino acid substitutions. However, all substitutions were situated outside of the canonical-binding groove.

Overall, the AltAll and ML PDZ3 domains showed a similar binding affinity for Eukaryota and native CRIPT (supplementary figs. 6 and tables 1 and 2, Supplementary Material online). Furthermore, secondary structure monitored by circular dichroism was similar for ML and AltAll variants, showing that the folding is robust (supplementary fig. 8*A*–*E*, Supplementary Material online). We determined the global stabilities by urea denaturation for all AltAll variants (supplementary fig. 8*F*–*J*, Supplementary Material online and table 3) and found that they were similar to the corresponding ML variants for 1R, bilaterians, and protostomes. The AltAll PDZ3 variant for the ancestor of deuterostomes displayed slightly lower stability compared with the ML variant (supplementary fig. 8*J*, Supplementary Material online and table 3). The curve fitting of AltAll and ML PDZ3 from the ancestor of hexapods suffered from sloping baselines and a short denatured baseline resulting in poorly defined *m*_D-N_ values (supplementary fig. 8*H*, Supplementary Material online). However, qualitatively, it is clear that the AltAll variant is less stable than ML, but still well folded. Thus, all AltAll variants were properly folded as judged by the circular dichroism spectra (supplementary fig. 8*A*–*E*, Supplementary Material online) and shape of the urea denaturation curves (as reflected in the *m*_D-N_ values) (supplementary fig. 8*F–J*, Supplementary Material online and table 3).

In conclusion, the AltAll and ML proteins displayed similar properties with regard to stability and binding, and therefore, other proteins in the ensemble of likely variants will most probably do that too, including the true ancestral PDZ3 variants (Eick et al. 2017).

### Neuroligin with Type 1 PBM Is Common among Deuterostome but Not Protostome Animals

The low affinity of the PDZ3:CRIPT interaction in nematodes and insects inspired us to look at another proposed biological ligand of DLG4 PDZ3, namely neuroligin. Like CRIPT, neuroligin interacts with PDZ3 via its C-terminus at synaptic junctions (Irie et al. 1997; Jeong et al. 2019). To compare the PBMs of neuroligin to those of CRIPT, the NCBI protein sequence database was searched for homologs of *Ho. sapiens* (UniProt: Q8N2Q7) and *C. elegans* (UniProt: Q9XTG1) neuroligin-1, as described for CRIPT in Materials and Methods section. *Ho. sapiens* and *C. elegans* neuroligin sequences were used for the search, because they were both of high quality and represented neuroligin sequences from deuterostomes and protostomes, respectively. The search resulted in 1,725 sequences in 437 species—419 metazoan species and 18 fungal species. Among the neuroligin sequences, type 1 PBM, like the one of CRIPT, was found to be predominant in deuterostomes, whereas type 2 PBM (ϕ-X-ϕ-COOH) was predominant in protostomes, especially hexapods (supplementary fig. 9, Supplementary Material online). Thus, we observe an even more dynamic evolution of the PBM in neuroligin as compared with CRIPT. It appears likely that bilaterian neuroligins recognize different PDZ domains in different species, whereas most of the neuroligin sequences from fungi and cnidarians—the only species outside of bilaterians that have neuroligin homologs—lack a PBM. However, the lower number of high-quality neuroligin sequences makes these results less conclusive than for CRIPT.

## Discussion

We have used ancestral sequence reconstruction and resurrection to better understand the connection between structure, function, and evolution of the PDZ3:CRIPT interaction and its biological function. Already at the time of the last common ancestor of all extant metazoa, CRIPT contained a type I PBM with high affinity (2–3 µM) to most present-day DLG PDZ3 domains, and likely to many other PDZ domains with type I PBM specificity. This affinity has remained constant or slightly increased (0.4–3 µM) among diverging lineages until today. But, at the beginning of the evolutionary lineage leading to present-day arthropods (∼550–600 My) a point mutation in the C-terminal residue of CRIPT resulted in substantially decreased affinity for DLG PDZ3, and again, likely all PDZ domains with type I specificity. What is the role of CRIPT and how would this mutation influence function? Large regions of CRIPT are highly conserved among extant species (supplementary fig. 10, Supplementary Material online) and mutation in CRIPT is very uncommon among humans, and associated with rare Mendelian disorders (Leduc et al. 2016). A recent study including in vivo experiments indicated an essential role for CRIPT in dendritic growth (Zhang et al. 2017). Experiments were performed in the nematode *C. elegans*, which, like *Dr. melanogaster*, has a Thr at the C-terminus of CRIPT. A severely mutated CRIPT (deletion of around 130 residues) resulted in abnormalities in dendritic growth, which could be rescued by expression of *Ho. sapiens* CRIPT. It was not possible to knock down CRIPT in mouse, suggesting a nonredundant and critical cell biological function in vertebrate species (Zhang et al. 2017). On the other hand, it has been possible to knock down one or several of the four DLG family members because these proteins can compensate for each other (Howard et al. 2010; Bonnet et al. 2013). In light of their experiments, and the lack of a type I PBM in the C-terminus of CRIPT from *C. elegans* and *Dr. melanogaster*, Zhang et al. (2017) suggested that the critical biological action of CRIPT could be partially independent of the PDZ3:CRIPT interaction in these animals. In the present article, we corroborate this idea by showing that PDZ3 domains from extant species of nematode and insect phyla bind their native CRIPT with significantly lower affinities than ancestral PDZ3:CRIPT pairs and those from extant vertebrates, even when CRIPT contains a large hydrophobic residue at the C-terminus, such as Leu in *L. loa* CRIPT. (There are also no obvious noncanonical internal PBMs in CRIPT.) The fact that PDZ3 domains from nematodes and insects have retained the affinity for type I PBM (*K*_d_=0.7–1.6 µM) reinforces the notion that the PDZ3:CRIPT interaction is nonfunctional in these species.

By necessity, many protein interaction studies are performed with domains cut out from highly modular, often large proteins, which are hard to work with in pure form. However, effects from regions outside the binding surface or domain are increasingly considered in protein–protein interaction studies. Indeed, extensions of PDZ domains are associated with several potential functions: 1) protein dynamics-based modulation of target-binding affinity, 2) provision of binding sites for macromolecular assembly, 3) structural integration of multidomain modules, and 4) expansion of the ligand-binding pocket (Wang et al. 2010). We analyzed the α_3_ extension in PDZ3 that possibly extends the ligand-binding surface beyond the canonical-binding groove. Interestingly, although this α_3_ extension had only minor effects on the low-affinity native PDZ3:CRIPT interactions (*L. loa*, *Dr. melanogaster*, and *De. ponderosae*), it indeed had a significant effect on binding to the high-affinity ancestral eukaryote CRIPT peptide to *L. loa* PDZ3, demonstrating high sequence dependence in the interaction, upon addition of α_3_. The sequence dependence was also reflected in the thermodynamic stabilities of PDZ3 with and without α_3_, where only *L. loa* PDZ3 displayed a clear stabilization in presence of the helix. With this in mind, we note that PDZ3 stabilities vary across present-day animals and that ancestral versions of PDZ3 were likely a little more stable. It may be assumed that random mutations, neutral for function, generally lead to a decrease in stability until a functional threshold is reached. It is therefore tempting to speculate that the relatively high stability (>6 kcal mol^−1^) of several ancestral and modern DLG4 PDZ3 domains is the result of positive selection.

Relatively few studies have used evolutionary biochemistry to assess protein–protein interactions (Wouters et al. 2003; Anderson et al. 2016a; Wheeler et al. 2018) and in particular attempting to reconstruct both interacting proteins (Hultqvist et al. 2017; Jemth et al. 2018). Wheeler et al. (2018) found that the extant S100A5 and S100A6 proteins display distinct specificity profiles toward a panel of peptide ligands and that this specificity was established early on during their respective evolutionary trajectory. However, the ancestral S100A5/A6 was promiscuous with regard to the tested peptide ligands. We observed a similar scenario for the interaction between two protein domains, NCBD and CID, from the transcriptional coregulators CBP/p300 and NCOA, respectively (Hultqvist et al. 2017). An ancestral NCBD domain displayed low affinity toward ancient and modern CIDs (but it displayed present-day affinities for other protein ligands). However, at the time point where we could reconstruct contemporary NCBD and CID (440 My), the interaction had the same affinity as modern complexes. These findings corroborate the hypothesis that protein interactions evolve toward higher specificity (Wheeler et al. 2016), but it is clear that “functional affinity” likely evolves relatively fast, in particular when it involves short disordered interaction motifs of three to ten residues, like for PDZ3:CRIPT. The affinity is then maintained by positive selection as long as it provides a benefit, or is lost by purifying selection if it is disadvantageous.

Evolutionary change of affinity and specificity is expected when new interactions arise and mutational equilibrium is likely established on a relatively short evolutionary time scale. For PDZ3, we do not observe the initial optimization of the affinity since the interaction is likely older than our analysis. The most ancient contemporary PDZ3:CRIPT pair we could reconstruct (Bilateria, 550–600 My) displays a 1.3–1.6 µM affinity. The *K*_d_ values of modern PDZ3:CRIPT pairs with a derived Thr at position −2 are slightly lower (0.4–0.5 µM) and might reflect adaptation toward increased specificity for Thr over Ser at position −2 (fig. 5). Although any functional advantage of the increase in specificity from the bilaterian ancestor is speculative, it is clear that affinity for the CRIPT PBM was lost in several protostome lineages by substitution of a Val for Ala or Thr at position 0 in CRIPT in an ancestral arthropod. Or even more curiously, by adaptations in PDZ3 as observed in the nematode lineage for *L. loa* (fig. 5 andsupplementary fig. 1, Supplementary Material online). Finally, an interesting notion from a structure-function point of view is that not only *Hy. vulgaris* (native interaction) but also human PDZ3 binds CRIPT with a C-terminal Cys residue equally well as CRIPT with the native Val. Clearly, affinity is not everything; A Cys residue can be easily modified and therefore might impose a disadvantage at this position and be subjected to purifying selection.

## Materials and Methods

### Phylogenetic Reconstruction

To collect protein sequences, the NCBI database (NCBI Resource Coordinators) was searched for CRIPT and DLG sequences using search terms and the BLAST tool (Altschul et al. 1990) with the *Ho. sapiens* DLG4 PDZ3 sequence. Sequences were then aligned using Clustal Omega software (Sievers et al. 2011) and filtered. Nonredundant sequences with information on species of origin, homologous to *Ho. sapiens* DLG4 PDZ3 or *Ho. sapiens* CRIPT, respectively, were kept. Sequences were filtered for redundancy, and, in the case of identical sequences among related species, they were combined into one sequence identifier. The final sets of sequences were aligned using Clustal Omega and manually curated. The resulting alignments were used to infer the ancestral sequences of DLG family PDZ3 domains and of CRIPT. For this sequence reconstruction, species trees were downloaded using the CommonTree tool of the Taxonomy Browser in the NCBI database. The taxonomy trees were modified before the ancestral reconstruction due to software requirements—the software could only read a tree that has exactly two branches per node, and the downloaded taxonomic trees sometimes had several branches per node, usually among species of the same genus. CommonTree does not provide branch lengths, but these are calculated by RAxML for the supplied tree during ancestral reconstruction. The PDZ3 tree was adjusted according to DLG family homology; due to evidence of genome duplication in early chordates, vertebrate sequences were split into DLG1, DLG2, DLG3, and DLG4 clades (McLysaght et al. 2002; Putnam et al. 2008). In the resulting CRIPT and PDZ3 trees, species names were replaced with sequence names to match the identifiers in the alignment. The RAxML software (Stamatakis 2014) was used to infer ancestral sequences for PDZ3 and CRIPT. Because of low posterior probabilities for some positions in the reconstructed ML sequences (supplementary data excel file 1, Supplementary Material online), we reconstructed AltAll sequences for each ML sequence. In the AltAll sequences, the amino acid residues with the second-highest probabilities were included for each position where the probability of the ML residue was <0.8.

Subsequently, we learned that RAxML version 8.0.0 implemented ancestral sequence reconstruction methods differently to other phylogeny software (Stamatakis A, Kozlov A, personal communication). Therefore, the ancestral sequence reconstruction was repeated using the newer RAxML-NG (Kozlov et al. 2019) and also with PAML (Yang 2007) software. Due to possible phylogenetic inaccuracies in the taxonomic tree, these programs were also tested with a second tree from OpenTree tree of life (Redelings and Holder 2017).

Overall, CRIPT ancestors reconstructed by RAxML-NG and PAML and using both trees were consistent with the RAxML-inferred ancestors, with some minor deviations (supplementary data excel file 2, Supplementary Material online). There was a variation between K/R in P_−4_ and S/T in P_−2_, and these conservative substitutions are unlikely to modulate binding, so the RAxML-inferred sequences were deemed valid for the analysis. The same analysis was carried out with PDZ sequences. For most PDZ sequences, amino acids in all structural and functional positions matched the originally used sequences. However, the ancestral deuterosomal PDZ3 sequence resurrected using PAML and RAxML-NG differed from the RAxML reconstruction in potentially crucial residues. This sequence was therefore expressed, purified, and subjected to experiments, where we demonstrated that binding and stability of the PAML- and RAxML-predicted variants were similar (supplementary fig. 5, Supplementary Material online).

For sequence similarity calculations, we used a matrix developed by Stephenson and Freeland (2013).

### Protein Expression and Purification

cDNA encoding DLG PDZ3 from the following extant species were cloned into a modified pRSET vector (Invitrogen) and transformed into *Escherichia coli* BL21(DE3) pLys cells (Invitrogen) for expression: *Ho. sapiens* (DLG4), *S. kowalevskii*, *Dr. melanogaster*, *De. ponderosae*, *L. loa*, *O. bimaculoides*, and *Hy. vulgaris*. Similarly, reconstructed cDNA encoding the following ancestral DLG PDZ3 (both ML and AltAll variants) were expressed: 1R, Deuterostomia, Bilateria, Hexapoda, and Protostomia. The expression construct contained an N-terminal 5xHis-tag. Cells were grown in LB medium at 37 °C and overexpression of protein was induced with 1 mM isopropyl-β-d-thiogalactopyranoside overnight at 18 °C. Cells were harvested by centrifugation at 4 °C and the pellet resuspended in 50 mM Tris, pH 7.8, 10% glycerol, and stored at 20 °C. Pellets were thawed and sonicated 2×4 min followed by centrifugation. The supernatant was filtered and added to a pre-equilibrated (50 mM Tris, pH 7.8, 10% glycerol) Nickel Sepharose Fast Flow column (GE Healthcare). Proteins were eluted with 250 mM imidazole and dialyzed into 50 mM Tris, pH 7.8, 10% glycerol buffer overnight. Proteins were then loaded onto a Q sepharose column for further purification and eluted with a 500 mM NaCl gradient in 50 mM Tris, pH 7.8, 10% glycerol. Protein purity and identity were analyzed by SDS–PAGE and MALDI-TOF mass spectrometry.

### Circular Dichroism and Urea Denaturation Experiments

Experiments were performed in 50 mM sodium phosphate, pH 7.45, 21 mM (*I *=* *150) at 10 °C on a JASCO J-1500 spectrapolarimeter. Far-UV spectra for all PDZ3 domains were recorded from 260 to 200 nm with 10 µM protein (the average of 16 scans). Thermodynamic stability of PDZ3 domains was determined by urea denaturation (0–8.1 M with 0.3 M steps) and monitored by molar ellipticity at 222 nm with 22 µM PDZ3. Data were fitted to a two-state unfolding process using both free fitting of each parameter or a shared *m*_D-N_ to obtain the midpoint (denoted [Urea]_50%_, where 50% of the protein is folded).

$$F=\frac{(\alpha_{N}+\beta_{N}\cdot \left[\mathrm{Urea}\right])+(\alpha_{D}+\beta_{D}\cdot \left[\mathrm{Urea}\right])\cdot e^{\frac{m_{D-N}\cdot {\left(\left[\mathrm{Urea}\right]-[\mathrm{Urea}\right]}_{50\%})}{R\cdot T}}}{1+e^{\frac{m_{D-N}\cdot {\left(\left[\mathrm{Urea}\right]-[\mathrm{Urea}\right]}_{50\%})}{R\cdot T}}},$$

where *F* is the observed spectroscopic signal; *α*_N_ is the signal of the native state in 0 M urea; *β*_N_ is the slope of the native baseline; and *α*_D_ and *β*_D_ are the corresponding parameters for the denatured state (Clarke et al. 1993).

### Kinetic Experiments

All kinetic experiments were performed in 50 mM sodium phosphate, pH 7.45, 21 mM KCl (*I* = 150) at 10 °C. Binding and dissociation experiments were performed at SX-17 MV stopped-flow spectrometer (Applied Photophysics) and carried out as described previously (Chi et al. 2010). Briefly, the association rate constant (*k*_on_) was obtained from binding experiments. PDZ3 (1 µM) was mixed rapidly with dansyl labeled (D) CRIPT peptide (concentration range: 2–20 µM). Excitation was done at 345 nm and emission was recorded >420 nm using a long-pass filter. The change in fluorescence signal obtained from D-CRIPT was monitored over time and fitted to a single exponential function to deduce the observed rate constant (*k*_obs_). Observed rate constants were plotted against the corresponding D-CRIPT concentration. Data were fitted to an equation for a reversible bimolecular association reaction (Malatesta 2005) to extract the association rate constant without considering pseudo first-order conditions.

$$k_{\mathrm{obs}}=\{k_{\mathrm{on}}^{2}{\left([\mathrm{PDZ}\right]}_{0}-{\left[\mathrm{CRIPT}\right]}_{0}{)}^{2}+k_{\mathrm{off}}^{2}+2k_{\mathrm{on}}k_{\mathrm{off}}([{\mathrm{PDZ}]}_{0}+{\left[\mathrm{CRIPT}\right]}_{0}+[{\mathrm{PDZ}{]}_{0})\}}^{0.5}$$

The dissociation rate constant (*k*_off_) was obtained from a separate displacement experiment. An equilibrium complex was preformed between PDZ3 and D-CRIPT (2:10 µM) and mixed rapidly with high excess of unlabeled CRIPT (100, 150, and 200 µM, respectively). The change in fluorescence signal obtained from dansyl-labeled CRIPT was monitored over time and fitted to a single exponential function to deduce the observed rate constant (*k*_obs_). The average of the three *k*_obs_ values is reported as the dissociation rate constant *k*_off_.

### Isothermal Titration Calorimetry Experiments

All isothermal titration calorimetry (ITC)-binding experiments were performed in 50 mM sodium phosphate pH 7.45, 21 mM KCl (*I* = 150) at 25 °C on an iTC200 microcalorimeter (Malvern Instruments). PDZ3 and CRIPT peptide were dialyzed against the same buffer to minimize artifacts from buffer mismatch in the titrations. CRIPT peptide was titrated 16 times into the cell containing PDZ3. For every titration, the heat release decreased from the binding of CRIPT to PDZ3, due to complex formation and PDZ3 saturation. Change in heat over time was integrated to kcal mol^−1^ and fitted to a one-site binding model to obtain binding stoichiometry (*n*), equilibrium binding constant (*K*_d_), and the enthalpy change upon binding (Δ*H*).

## Data Availability

The authors declare that data supporting the findings of this study are available within the article and its supplementary information file, Supplementary Material online, including probability tables for reconstruction of CRIPT and PDZ3 sequences. Additional raw data are available from corresponding authors upon request.

## Supplementary Material

Supplementary data are available at *Molecular Biology and Evolution* online.

## Supplementary Material

msaa198_Supplementary_Data
